# Cardiac Fibrosis Is a Risk Factor for Severe COVID-19

**DOI:** 10.3389/fimmu.2021.740260

**Published:** 2021-10-22

**Authors:** Julian Mustroph, Julian Hupf, Maria J. Baier, Katja Evert, Christoph Brochhausen, Katharina Broeker, Christine Meindl, Benedikt Seither, Carsten Jungbauer, Matthias Evert, Lars S. Maier, Stefan Wagner

**Affiliations:** ^1^ Department of Internal Medicine II, University Hospital Regensburg, Regensburg, Germany; ^2^ Emergency Department, University Hospital Regensburg, Regensburg, Germany; ^3^ Institute of Pathology, University of Regensburg, Regensburg, Germany; ^4^ Department of Physiology, University of Regensburg, Regensburg, Germany

**Keywords:** SARS-CoV-2, COVID-19, fibrosis, heart, TGF-β1, NRP-1

## Abstract

Increased left ventricular fibrosis has been reported in patients hospitalized with coronavirus disease 2019 (COVID-19). It is unclear whether this fibrosis is a consequence of severe acute respiratory syndrome coronavirus type 2 (SARS-CoV-2) infection or a risk factor for severe disease progression. We observed increased fibrosis in the left ventricular myocardium of deceased COVID-19 patients, compared with matched controls. We also detected increased mRNA levels of soluble interleukin-1 receptor-like 1 (sIL1-RL1) and transforming growth factor β1 (TGF-β1) in the left ventricular myocardium of deceased COVID-19 patients. Biochemical analysis of blood sampled from patients admitted to the emergency department (ED) with COVID-19 revealed highly elevated levels of TGF-β1 mRNA in these patients compared to controls. Left ventricular strain measured by echocardiography as a marker of pre-existing cardiac fibrosis correlated strongly with blood TGF-β1 mRNA levels and predicted disease severity in COVID-19 patients. In the left ventricular myocardium and lungs of COVID-19 patients, we found increased neuropilin-1 (NRP-1) RNA levels, which correlated strongly with the prevalence of pulmonary SARS-CoV-2 nucleocapsid. Cardiac and pulmonary fibrosis may therefore predispose these patients to increased cellular viral entry in the lung, which may explain the worse clinical outcome observed in our cohort. Our study demonstrates that patients at risk of clinical deterioration can be identified early by echocardiographic strain analysis and quantification of blood TGF-β1 mRNA performed at the time of first medical contact.

## Introduction

During the 2020 coronavirus disease 2019 (COVID-19) pandemic, direct cardiac manifestations of infection with severe acute respiratory syndrome coronavirus type 2 (SARS-CoV-2), such as heart failure, arrhythmias (1–3), and general ischemic events, were increasingly detected (4, 5). Myocardial magnetic resonance imaging (MRI) of patients with COVID-19 and patients who were post-recovery revealed increased levels of myocardial fibrosis, particularly in patients who had experienced severe COVID-19 disease courses (6, 7). It is currently unknown whether cardiac fibrosis is caused by SARS-CoV-2 infection or whether pre-existing fibrosis is a risk factor for severe courses of COVID-19.

Cardiac fibrosis is induced by a myriad of cardiac diseases, including myocardial infarction, heart failure and chronic arrhythmias. A hallmark of its development is increased fibroblast activation, which is caused by increased levels of transforming growth factor β1 (TGF-β1) (8).

Interleukin-33 (IL-33) is the ligand for interleukin-1 receptor-like 1 (IL1-RL1, also known as ST2), a receptor expressed on the cell surface of fibroblasts and cardiomyocytes (9–11). The interaction of IL-33 with IL1-RL1 is regulated by cardiomyocyte stretch, which exerts anti-inflammatory, anti-fibrotic, and anti-hypertrophic effects physiologically. Cardiac injury and fibroblast activation *via* TGF-β1 induce the soluble form of interleukin-1 receptor-like 1 (sIL1-RL1 [also known as sST2]), which serves as a decoy receptor for IL-33 and inhibits the cardioprotective effects of IL-33/IL1-RL1 interaction (9, 10, 12).

Increased levels of sIL1-RL1 can also induce neuropilin-1 (NRP-1) expression, a co-receptor for TGF-β1 (13, 14). As increased levels of circulating and local TGF-β1 and sIL1-RL1 are a hallmark of cardiac fibrosis (15, 16), and increased sIL1-RL1-induced NRP-1 expression has been demonstrated in rat cardiomyocytes and human cardiac fibroblasts (12), it is reasonable to infer that NRP-1 could also be increased in human fibrotic hearts.

The novel coronavirus SARS-CoV-2 has been shown to bind to host cells *via* its spike protein, using the enzymatic domain of angiotensin-converting enzyme 2 (ACE2) to enter the cell (17).

Recently, it has been shown that NRP-1 facilitates SARS-CoV-2 entry into the host cell (18). We therefore hypothesized that patients with cardiac fibrosis might be at risk for severe COVID-19 progression owing to increased NRP-1 expression in the heart and lung.

## Methods

### Patient Recruitment and Classification of Disease Severity

The study was approved by the ethics committee at the University of Regensburg (statement 20-1765-101). All investigations conformed to the ethical principles that have their origin in the Declaration of Helsinki. Written informed consent was obtained from all patients prior to study inclusion.

Patients were enrolled if they presented to the emergency department (ED) with signs of acute respiratory infection. Study inclusion criteria were age ≥ 18 years and signs of acute respiratory infection indicative of COVID-19. Since all SARS-CoV-2-positive patients had symptomatic disease, the term COVID-19 is used synonymously for these patients. Exclusion criteria were patients incapable of freely providing informed consent and any delay of life-saving diagnostic or therapeutic measures that would be caused by study involvement. In this study, we first included patients with suspected COVID-19, defined by respiratory infection with or without reported fever. Patients were then tested for SARS-CoV-2 infection (see below). The patients with a negative SARS-CoV-2 test result who presented with viral or bacterial respiratory infection were used as a control group. The exact cause of infection in non-SARS-CoV-2 respiratory tract infection could not be ascertained, but we were able to exclude influenza because it was routinely tested in the ED.

After consent, clinical baseline characteristics and vital signs were documented for each patient. Blood was drawn by venipuncture from each patient immediately after admission to the ED and the potentially virus-containing blood samples were inactivated using Trifast (Ambion). Echocardiography was performed in a subset of patients, typically within two hours after ED admission. Furthermore, outcome and complications during the hospital stay were monitored. Severe disease (“SD”) was defined as the subsequent need for mechanical ventilation, admission to an intensive care unit (ICU) or death. Otherwise, patients were classified as exhibiting moderate disease (“MD”).

### Quantitative Reverse Transcriptase Polymerase Chain Reaction (RT-PCR) From Whole Blood

Blood drawn from patients after admission was used for quantitative PCR (qPCR) of the markers specified below. Whole-blood analysis was chosen for its practicability, requiring no cell or plasma separation stages, and also for safety reasons, as SARS-CoV-2 can be inactivated easily using Trifast without opening the blood tubes, thus avoiding potentially hazardous aerosol generation. The investigators involved in qPCR analysis were blinded to group assignment/SARS-CoV-2 test status. Since some patients with already verified SARS-CoV-2 infection were referred to our hospital ED by primary care physicians, for safety reasons the nurses and physicians who drew blood and recorded clinical data could not always be blinded to SARS-CoV-2 infection status. Nevertheless, all patients included in the study were treated equally in terms of diagnostic testing, timing of testing, and safety precautions.

Quantitative RT-PCR was performed as described elsewhere (19, 20). Briefly, RNA was extracted using trichloromethane-chloroform solution and isopropranolol solution. RNA was purified using the RNeasy Plus Mini Kit (Qiagen) and reverse transcribed to cDNA. The following primers (all by Applied Biosystems, catalogue numbers in parentheses) were used to analyze cDNA on Taqman PCR apparatus: TGF-β1 (Hs00998133_m1), SMAD3 (Hs00969210_m1), NOX2 (Hs00166163_m1), NOX4 (Hs01379108_m1), sIL1-RL1 (Hs00249384_m1), and NRP-1 (Hs00826128_m1). Expression was normalized to either β-actin or GAPDH (β-actin: Hs00357333_g1, GAPDH: Hs02786624_g1).

### Tissue Acquisition and Macroscopic Analysis

Left ventricular cardiac tissue from patients who died of COVID-19 was obtained during autopsies, after written informed consent had been provided by the next of kin.

For ethical and legal reasons, these deceased patients were not all from our ED blood study. All patients exhibited symptomatic disease and SARS-CoV-2 infection that had been verified by PCR from respiratory material, which was determined to be the cause of death. Owing to the scarcity of control tissue, we used a combination of left ventricular tissue taken from patients who had died from other respiratory infections and from patients whose hearts had been destined for donation that ultimately could not be performed. Remarkably, there was no difference between the entities that made up the control group.

After removal of the heart in toto, a macroscopic evaluation was performed according to standard operational procedure. Macroscopically conspicuous regions, i.e. with color changes, hyperemic areas, areas with signs of accompanying pericarditis (fibrin deposits), were histologically processed and examined. If no macroscopic abnormalities were present, tissue sections were obtained from each cardiac region (left atrium, right atrium, right ventricle, septum, lateral wall of left ventricle) for further pathological analysis.

### Immunohistochemistry

4 µm thick tissue sections with an area of approximately 1x1 cm were prepared from paraffin-embedded hearts (left ventricle) and lungs and fixed on slides. Adjacent sections were stained with trichrome and antibody directed against CD3. Trichrome staining was performed in left ventricular sections using a trichrome kit (HT15, Sigma-Aldrich) according to the manufacturer’s protocol. Briefly, deparaffinized and rehydrated sections were incubated in Bouin’s Solution overnight (at RT). Sections were subsequently stained with Weigert’s iron hematoxylin for 5 min, with Biebrich acid fuchsin for 4 min and with aniline blue for 12 min. Immunohistology for CD3 (catalogue number A0452, Dako, 1:200 in PBS) was performed using an automated immunostainer (Ventana Benchmark Ultra, Roche Diagnostics) according to the manufacturer’s instructions.

For NRP-1, after blocking with an Avidin/Biotin Blocking Kit (SP-2001, Vector Laboratories) and SuperBlock™ Blocking Buffer (37515, Thermo Fisher Scientific) for 30 min, lung sections were single stained with a rabbit anti-human neuropilin 1 antibody (ab81321, abcam, 1:200 in PBS) overnight (4°C). Biotin donkey anti-rabbit IgG (711-065-152, Jackson ImmunoResearch, 1:250 in PBS, 1h at room temperature) were used as secondary antibody. Afterwards sections were incubated with VECTASTAIN Elite ABC Kit (Vector PK-6100, 30 min) and stained with DAB solution (5 min, 37°C).

Images of whole, trichrome- or NRP-1-stained tissue sections were captured with a Zeiss Axiostar plus microscope (Carl Zeiss Microscopy GmbH, 10x magnification). Image analysis was performed with HistoQuest Software to calculate the proportion of NRP-1 or collagen-stained area (in %) relative to the total surface area. For analysis of CD3+ cells, 50 images (= FOVs, fields of view) per section were recorded with a Zeiss Axiostar plus microscope (Carl Zeiss Microscopy GmbH, 20x magnification) and the mean number of CD3+ cells/FOV was calculated.

### Immunofluorescence Microscopy

Before staining, pulmonary sections were deparaffinized and rehydrated (2x 10 min in xylol, 2x 5 min in 100% EtOH, 2x 5 min in 96% EtOH, 2x 5 min in 70% EtOH, 5 min in PBS). Antigen retrieval was performed by boiling for 30 s at 121°C in *Antigen Unmasking Solution* (catalogue number H-3300-250, Vector Laboratories, 1:100 in dH_2_O).

After blocking with SuperBlock™ Blocking Buffer (catalogue number 37515, Thermo Fisher Scientific) for 1h, lung sections were incubated overnight at 4°C with mouse anti-human SARS/SARS-CoV-2 (catalogue number MA1-7404, Invitrogen, 1:100 in PBS) and rabbit anti-human NRP-1 (catalogue number ab81321, abcam, 1:200 in PBS) antibodies.

An Avidin/Biotin Blocking Kit (catalogue number SP-2001, Vector Laboratories) was used before incubation with rabbit anti-human NRP-1 primary antibody. Streptavidin Alexa Fluor 594 (catalogue number 016-580-084, Jackson ImmunoResearch, 1:250 in PBS) and goat anti-rabbit IgG Alexa Fluor 488 (catalogue number A11008, Invitrogen, 1:500 in PBS) were used as secondary antibodies, incubation time was 1 h at room temperature.

Nuclei were visualized by staining with Hoechst (catalogue number 33342, Molecular Probes, 1:50.000 in PBS, 2 min). To quench autofluorescence, sections were incubated for 2 min with Vector^®^ TrueVIEW^®^ Autofluorescence Quenching Kit (catalogue number SP-8400-15, Vector Laboratories).

Images of lung sections labelled with immunofluorescence for SARS-CoV-2/NRP-1 were recorded with a Zeiss LSM7 confocal microscope (Carl Zeiss Microscopy GmbH, 40x magnification).

The region of interest (ROI, typically about a third of the whole image area) was set to contain as much tissue as possible, avoiding large alveolae (i.e. non-stained areas). For each channel, nonspecific fluorescence was eliminated by setting a minimal fluorescence threshold; the resulting black pixels were set to “not a number” (NaN) so that tissue-free areas were automatically excluded from the analysis. The mean ROI fluorescence intensity was then calculated.

### Western Blots

Western blots were performed to confirm key results, as described elsewhere (21). Briefly, left ventricular myocardium was mechanically homogenized (using a stainless steel pestle) in Tris buffer containing (in mmol/L): 50 Tris-HCl, 200 NaCl, 20 NaF, 1 Na_3_VO_4_, 1 DTT [pH 7.4] and protease inhibitor cocktail. Protein concentration was determined by BCA assay. Proteins were denaturated for 5 minutes at 95 °C (for analysis of CaMKII and ox-CaMKII) or for 30 minutes at 37 °C (for analysis of NRP-1) in 2% β-mercaptoethanol. To avoid unspecific reduction of oxidized CaMKII, β-mercaptoethanol was omitted in the lysates for ox-CaMKII. Proteins were separated on 8% sodium dodecyl sulfate polyacrylamide gels, then transferred to a nitrocellulose membrane and incubated in Tris-buffered saline with tween-20 (TBS-T) with 5% milk at 4 °C overnight with the following primary antibodies: anti-CaMKII (Don Bers’ lab, 1:10 000), anti-ox-CaMKII (catalogue number GTX36254, GeneTex, 1:1000), anti-NRP-1 (catalogue number ab81321, Abcam, 1:1000) and anti-GAPDH (catalogue number G8795, Sigma-Aldrich, 1:10 000). The secondary antibodies HRP-conjugated donkey anti-rabbit and sheep anti-mouse IgG (catalogue numbers NA934 and NA931, GE Healthcare,1:10 000) were applied and incubated for 1 hour at room temperature. For chemiluminescent detection, WesternBright™ ECL HRP substrate (catalogue number K-12045-D50, advansta) was used. Protein was normalized to GAPDH using ImageJ to determine mean densitometry.

### SARS-CoV-2 Diagnostic Tests

PCR testing for SARS-CoV-2 at our institution mainly used pharyngeal rinse water, but we also accepted results from external facilities that had used throat swabs and sputum, if the test had been performed by a certified laboratory. All respiratory specimens were analyzed immediately after collection in the diagnostic virology unit of our hospital by means of a commercial IVD/CE-compliant RT-PCR assay (Xpert^®^ Xpress SARS-CoV-2, Cepheid GmbH, Krefeld, Germany).

All patients with a positive PCR test result displayed signs of respiratory infection, so the term “COVID-19” is used synonymously for all SARS-CoV-2-positive patients.

Hearts taken from deceased COVID-19 patients were analyzed for SARS-CoV-2 using real time PCR as previously described (22). Trials to replicate and thus enrich SARS-CoV-2 from patient tissue in permanent monolayer cell cultures followed by real time PCR could also not detect SARS-CoV-2 in heart tissue from our COVID-19 patient cohort. For PCR, nucleic acids were extracted from fresh-frozen organ slices using the EZ1 Virus Mini Kit v2.0 with the EZ1 Advanced XL system (Qiagen, Hilden, Germany). Viral ssRNA was amplified using the SARS-CoV-2 E gene RT-PCR with StepOnePlus Real-Time PCR System (Thermo Fisher Scientific, Schwerte, Germany). Bacteriophage MS2 served as an internal control for extraction and amplification efficacy.

### Statistics

Normality of data was tested by hierarchical application of D’Agostino-Pearson, Shapiro-Wilk, or Kolmogorov-Smirnov tests, respectively, depending on the number of experiments conducted. For data with normal distribution, a Student’s t-test was performed in the case of two groups without pairing. When testing multiple groups, an analysis of variance (ANOVA) was performed. For data for which normality could not be assumed, a Mann-Whitney test was performed on two groups without pairing. Otherwise, a Kruskal-Wallis test was used. The respective post-tests adjusting for errors for multiple testing are referenced in the figure legends. Categorical data were tested using the Fisher’s exact test. The significance level was taken at 5% (two-sided p). Data are presented as mean ± standard error of the mean (SEM), if not otherwise indicated. No data were excluded from the analysis.

Statistical analyses were performed using GraphPad Prism v9 (GraphPad Software, San Diego, CA, USA) and SPSS 26.

## Results

### Deceased COVID-19 Patients Display Increased Cardiac Fibrosis

To test whether patients who died from COVID-19 show increased left ventricular fibrosis, we obtained left ventricular autopsy sections from deceased patients. SARS-CoV-2 infection was detected by PCR in all of these patients. Patient characteristics of all control and COVID-19 patients can be found in **Table 1**. **Table 2** shows the characteristics of the patients for whom histological data were available. The deceased COVID-19 patients were of similar age compared to the controls. Patients were typically male, and patients who died of COVID-19 were more likely to be obese. Troponin has been reported to be elevated in severe cases of COVID-19 (1, 3), and this was also evident in our cohort.

**Table 1:** Patient characteristics (all patients).

	Control (n = 41)	Moderate COVID-19 (n = 23)	Severe COVID-19 (n = 13)	Statistics
**Baseline characteristics**				
**Age: mean ± SD (n)**	58.86 ± 17.85 (35)	47.35 ± 16.39 (23)	60 ± 11.06 (13)	p=0.021
**Sex: % male (n)**	63 (22)	48 (11)	62 (8)	p=0.5
**BMI: mean ± SD**	26.41 ± 5.26	26.93 ± 4.86	31.68 ± 7.91	p=0.019
**Diabetes: % (n)**	26 (9)	13 (3)	15 (2)	p=0.54
**Hypertension: % (n)**	34 (12)	26 (6)	62 (8)	p=0.094
**Coronary artery disease: % (n)**	34 (12)	4 (1)	23 (3)	p=0.024
**Baseline medication**				
**RAAS-inhibitors: % (n)**	34 (12)	22 (5)	15 (2)	p=0.4
**Beta receptor blockers:**	26 (9)	0 (0)	69 (9)	p<0.001
**Aspirin (100 mg/d): % (n)**	40 (14)	4 (1)	23 (3)	p=0.005
**Oral antidiabetics: % (n)**	23 (8)	13 (3)	8 (1)	p=0.53
**Insulin: % (n)**	9 (3)	9 (2)	0 (0)	p=0.7
**Steroids: % (n)**	26 (9)	0 (0)	0 (0)	p=0.004
**Admission**				
**Body temperature: °C, median (IQR; n)**	37.3 (36.6, 38.3; 31)	37.4 (37.1, 38.3; 23)	37.6 (37, 38.5; 13)	p=0.79
**LVEF (%): median (IQR; n)**	60 (50, 60; 29)	55 (53, 60; 18)	55 (46, 56.5; 12)	p=0.2
**AFib at admission: % (n)**	14 (5)	4 (1)	15 (2)	p=0.26
**Creatinine mg/dl, mean ± SD (n)**	1.27 ± 1.00 (33)	0.90 ± 0.29 (23)	3.00 ± 4.52 (12)	p=0.014
**NT-proBNP pg/ml, median (IQR) (n)**	528 (64.1, 1895) (26)	49 (49, 164) (23)	4349 (74.4, 9835) (10)	p<0.001
**CRP mg/l, mean ± SD (n)**	73.86 ± 70.70 (33)	33.55 ± 26.35 (23)	173.08 ± 113.68 (12)	p=0.21
**Hemoglobin g/dl, mean ± SD (n)**	11.33 ± 2.64 (33)	13.62 ± 1.17 (23)	11.15 ± 3.31 (12)	p=0.001
**Troponin (x upper norm), median (IQR) (n)**	0.88 (0.31, 4.04) (27)	0.39 (0.27, 0.64) (23)	2.85 (0.95, 4.02) (12)	p<0.001
**pH (venous blood gas), mean ± SD (n)**	7.41 ± 0.05 (26)	7.42± 0.06 (23)	7.39 ± 0.13 (6)	p=0.53
**Hospital stay**				
**Steroid treatment, % (n)**	11 (4)	0 (0)	62 (8)	p<0.001
**Reconvalescent plasma, % (n)**	0 (0)	0 (0)	46 (6)	p<0.001
**Intubation, % (n)**	20 (7)	0 (0)	85 (11)	p<0.001
**ECMO (vv or va), % (n)**	6 (2)	0 (0)	38 (5)	p=0.001
**Dialysis, % (n)**	6 (2)	0 (0)	69 (9)	p<0.001

IQR, interquartile range; BMI, body mass index; RAAS, renin-angiontensin-aldosteron-system; AFib, atrial fibrillation; CRP, c-reactive protein; ECMO, extracorporal membrane oxygenation; vv, veno-venous; va, veno-arterial.

**Table 2:** Patient characteristics (histological data).

	Control (n = 8)	Severe COVID-19 (n = 8)	Statistics
**Baseline characteristics**			
**Age: mean ± SD**	56 ± 15.01	61.88 ± 9.70	p=0.37
**Sex: % male (n)**	75 (6)	50 (4)	p=0.3
**BMI: mean ± SD (n)**	25.01 ± 5.85	31.25 ± 5.52	p=0.064
**Diabetes: % (n)**	25 (2)	12 (1)	p=1.0
**Hypertension: % (n)**	25 (2)	62 (5)	p=0.31
**Coronary artery disease: % (n)**	12 (1)	12 (1)	p=1.0
**Baseline medication**			
**RAAS-inhibitors: % (n)**	25 (2)	12 (1)	p=1.0
**Beta receptor blockers:**	12 (1)	62 (5)	p=0.12
**Aspirin (100 mg/d): % (n)**	38 (3)	12 (1)	p=0.57
**Oral antidiabetics: % (n)**	25 (2)	0 (0)	p=0.47
**Insulin: % (n)**	25 (2)	0 (0)	p=0.47
**Steroids: % (n)**	12 (1)	0 (0)	p=1.0
**Admission**			
**Body temperature: °C, median (IQR; n)**	36.8 (36.3, 37.2; 5)	37.2 (36, 38.35; 8)	p=0.88
**LVEF (%): median (IQR; n)**	58 (53, 60; 4)	55 (53, 60; 7)	p=0.56
**AFib at admission: % (n)**	0	12 (1)	p=0.47
**Creatinine mg/dl, mean ± SD (n)**	1.87 ± 1.24 (7)	1.96 ± 0.93 (8)	p=0.88
**NT-proBNP pg/ml, median (IQR; n)**	3390 (3390, 3390; 1)	6370 (1514, 21076; 6)	p=1.0
**CRP mg/l, mean ± SD (n)**	100.76 ± 69.31 (7)	158.8 ± 84.39 (8)	p=0.17
**Hemoglobin g/dl, mean ± SD (n)**	8.63 ± 2.49 (7)	10.08 ± 3.46 (8)	p=0.38
**Troponin (x upper norm), median (IQR; n)**	8.41 (2.53, 14.30; 2)	2.85 (0.95, 3.71; 8)	p=0.60
**Hospital stay**			
**Steroid treatment, % (n)**	12 (1)	100 (8)	p=0.001
**Reconvalescent plasma, % (n)**	0 (0)	50 (4)	p=0.077
**Intubation, % (n)**	75 (6)	100 (8)	p<0.001
**ECMO (vv or va), % (n)**	25 (2)	62 (5)	p=0.31
**Dialysis, % (n)**	25 (2)	88 (7)	p=0.47

IQR, interquartile range; BMI, body mass index; RAAS, renin-angiontensin-aldosteron-system; AFib, atrial fibrillation; CRP, c-reactive protein; ECMO, extracorporal membrane oxygenation; vv, veno-venous; va, veno-arterial.

**Figure 1:**
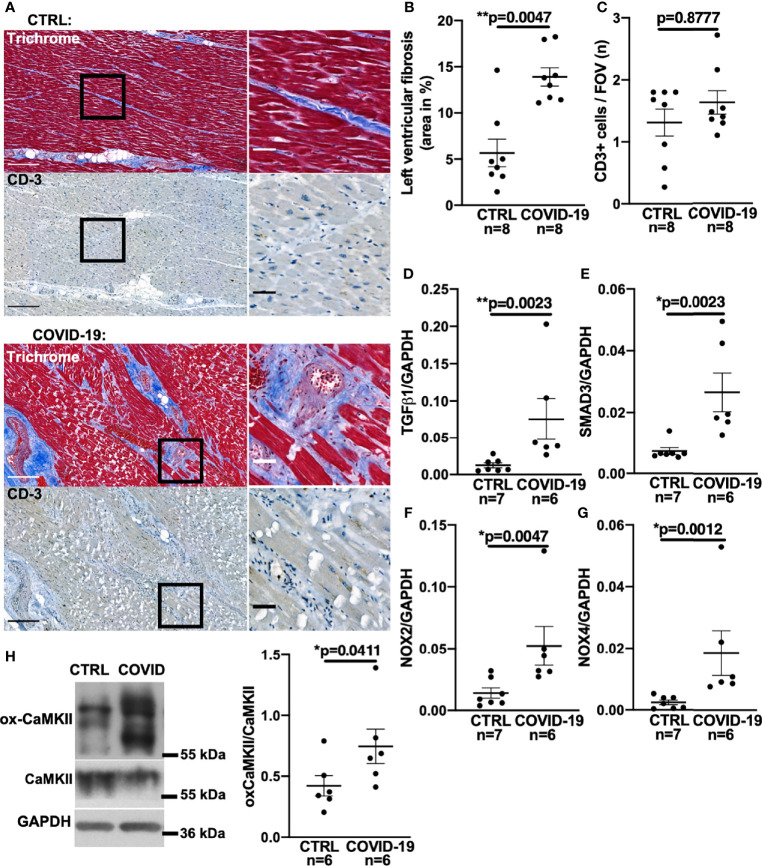
Increased left ventricular fibrosis in deceased patients with COVID-19. **(A)** Original histochemical stainings using trichrome (upper panels) and immunohistochemical stainings for CD3 (lower panels) of adjacent sections of left ventricular tissue. Left images with scale bar 250 µm. Right images show a magnified view of the areas marked with a solid black border (scale bar 50 µm). **(B)** Mean ± SEM data of left ventricular myocardial fibrosis area shows increased myocardial fibrosis in autopsied COVID-19 patients compared to control (CTRL; Mann-Whitney test). **(C)** Mean ± SEM number of CD3-positive cells per field of view (FOV) shows no significant difference between COVID-19 patients and controls (Mann-Whitney test). **(D–G)** Mean levels of mRNA expression of TGF-β1 **(D)**, SMAD3 **(E)**, NOX2 **(F)** and NOX4 **(G)** detected by qPCR in left ventricular myocardium taken from deceased COVID-19 patients and controls. Compared to controls, mRNA expression of TGF-β1, SMAD3, NOX2 and NOX4 was significantly increased in autopsied COVID-19 patients (all tested with Mann-Whitney). **(H)** Original western blot (left panel) and mean densitometric data (right panel) for oxidized CaMKII (ox-CaMKII) expression normalized to CaMKII. In myocardium of patients with COVID-19, oxidized CaMKII expression was significantly increased (Mann-Whitney test).

**Figure 1A** shows original trichrome stains and CD3-stainings of histological sections from left ventricular autopsies of a patient who died from COVID-19 (lower panel) and a control patient (upper panel). **Figure 1B** shows a significantly increased left ventricular fibrotic area quantified using trichrome staining in deceased COVID-19 patients compared to control. In a subset of these patients, echocardiography results showed no difference in left ventricular ejection fraction between control and COVID-19 patients (**Supplementary Figure 1A**). Interestingly, left ventricular fibrosis was significantly increased in deceased COVID-19 patients even when only the subgroup with available echo data was analyzed (**Supplementary Figure 1B**).

One cause of myocardial fibrosis could be persistent myocarditis. However, to date, histopathologically confirmed COVID-19-induced myocarditis has been shown to be rare (22, 23) and largely limited to patients under non-steroidal immunosuppression (23). In our cohort, no SARS-CoV-2 virus was detected in the left ventricular myocardium of the 8 deceased patients by PCR and cell culture assays. Furthermore, no signs of current or post-inflammatory viral myocarditis could be found by our pathologists. To test for signs of myocardial leukocyte infiltration, we additionally performed CD3 staining but could not detect significant differences between COVID-19 and control groups (original left ventricular CD3 staining in **Figure 1A**, mean data in **Figure 1C**). Thus, myocardial fibrogenesis due to viral myocarditis seems unlikely.

### Fibrotic Markers Are Increased in Deceased COVID-19 Patients

Myocardial fibrogenesis is induced by TGF-β1 (13, 15, 24–26). To test whether TGF-β1 is increased in deceased COVID-19 patients, we measured TGF-β1 mRNA levels by quantitative RT-PCR in left ventricular tissue taken from patients with COVID-19. **Figure 1D** shows significantly increased TGF-β1 RNA levels in left ventricular tissue from patients who died of COVID-19 compared with controls.

TGF-β1 binding to its receptor leads to phosphorylation of intracellular SMAD3, which upregulates profibrotic genes (15, 24, 25), resulting in increased expression of collagens or fibronectin. Although altered levels of SMAD3 RNA would not necessarily equate to altered SMAD3 activity, it has been shown in a variety of tissues that TGF-β1-induced upregulation of SMAD3 can be an important inducer of reactive oxygen species (ROS) *via* upregulation of NADPH oxidases II and IV (NOX2/NOX4) (25–29). We therefore measured SMAD3 in left ventricular tissue from deceased COVID-19 patients and found the mRNA levels to be significantly increased (**Figure 1E**).

NOX2- and NOX4-dependent ROS production (27, 30) can oxidize Ca^2+^/Calmodulin-dependent-kinase II (CaMKII), a serine-threonine kinase which regulates fibrosis. Oxidized CaMKII changes its conformation, thereby enabling phosphorylation of downstream targets (31). Oxidized CaMKII is also a marker of fibrosis (32) and inhibition of CaMKII has been shown to ameliorate the development of fibrosis (32).

Direct measurement of ROS-production in tissue autopsy samples is futile owing to the postmortem decay of ROS, but NOX RNA levels correlate strongly with ROS production (33). Accordingly, we demonstrate here increased levels of both NOX2 and NOX4 mRNA in left ventricular tissue from patients with COVID-19 (**Figures 1F, G**). Western blots of left ventricular myocardium from patients with COVID-19 show increased expression of the oxidized form of CaMKII normalized to total CaMKII expression, which provides further evidence of increased ROS production (**Figure 1H**).

### TGF-β1 Expression and Left Ventricular Strain Predict Moderate and Severe COVID-19 Disease Courses

Cardiac fibrosis typically develops over months or even years. It therefore seems highly plausible that the extensive fibrosis observed in our deceased COVID-19 patients did not develop during a few weeks of SARS-CoV-2 infection, but that existing fibrosis predisposes patients to severe disease progression.

We tested whether elevated TGF-β1 mRNA levels were already present in COVID-19 patients who were newly-admitted to our ED and presumably at an early stage of the disease, when relevant fibrosis due to infection is unlikely. For this purpose, whole blood was drawn from these patients immediately after admission and disease progression was subsequently monitored. COVID-19 patients who later required hospitalization were categorized as “moderate disease” (MD), while patients who later required intensive care with intubation or died were categorized as “severe disease” (SD).

**Figure 2:**
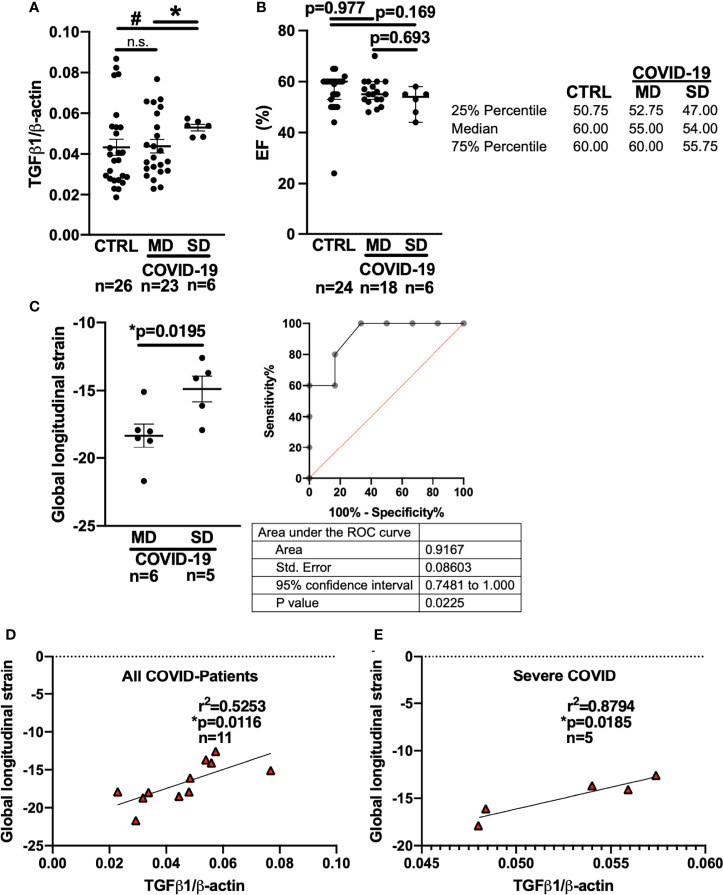
Left ventricular global longitudinal strain and whole blood mRNA levels of TGF-β1 can discriminate moderate and severe COVID-19 at first medical contact in the ED. **(A)** Mean ± SEM mRNA expression (qPCR) of TGF-β1 in blood drawn from newly hospitalized COVID-19 patients and controls (CTRL, other respiratory infections) in the ED. The clinical course of the COVID-19 patients was monitored and stratified into moderate (MD) or severe disease (SD). Patients who later developed SD had increased TGF-β1 mRNA levels already at the time of admission to the ED (Welsh ANOVA with two-stage linear step-up procedure of Benjamini, Krieger and Yekutieli, # p=0.0154 SD *vs.* CTRL, * p=0.0154 MD *vs.* SD). **(B)** Left ventricular ejection fraction (median ± 95% confidence interval, echocardiography performed in the ED). There was no significant difference between CTRL patients and COVID-19 patients with either MD or SD (Kruskal-Wallis test with Dunn’s post test). **(C)** Left panel: Mean ± SEM left ventricular global longitudinal strain, an echocardiographic marker of fibrosis. Left ventricular strain was significantly less negative in patients who later developed SD compared to patients who had developed MD already at the time of admission to the ED (Mann-Whitney test). Right panel: ROC curve of global longitudinal strain accurately predicts the subsequent development of SD already in the ED. **(D, E)** Correlation analysis (Pearson correlation) of global longitudinal strain with TGF-β1 mRNA expression (lines indicate linear regression). There was a significant correlation within all COVID-19 patients **(D)**, but especially in patients who would later develop SD **(E)**.

Interestingly, compared with patients who developed MD, patients who later developed SD had significantly higher levels of TGF-β1 mRNA in the blood at the time of initial presentation to the ED (**Figure 2A**). In addition, patients who later developed SD had significantly increased TGF-β1 mRNA levels compared with control patients (“CTRL”) exhibiting other viral or bacterial respiratory infections.

In eleven COVID-19 patients, we were able to perform echocardiography in the ED. Remarkably, there was no significant difference in left ventricular ejection fraction between the MD and SD groups at the time of admission to the ED (**Figure 2B**, Kruskal-Wallis-test). It is not ethically permissible or technically feasible to perform a left ventricular biopsy in patients with subsequent severe disease and especially moderate disease, in order to test for fibrosis. However, left ventricular global longitudinal strain, which measures the deformation of the cardiac wall during contraction, is an established indicator of cardiac fibrosis (34–36). In fibrotic hearts, ventricular global longitudinal strain is increased, i.e. produces a less negative numerical reading. Strain analysis requires co-acquisition of an electrocardiogram during echocardiography and high-quality recordings of the parasternal, four-chamber, and three chamber axes, which is not always possible in an emergency department setting.

Echocardiography recordings from 6 MD patients and 5 SD patients fulfilled the technical requirements for strain analysis, which was performed by a blinded core laboratory investigator who was not involved in patient care or study recruitment.

In the ED, left ventricular strain was already significantly increased in patients who later developed severe COVID-19 compared to patients who would later develop moderate disease (**Figure 2C**, Mann-Whitney-test). Interestingly, receiver operating characteristics (ROC) analysis showed that left ventricular global longitudinal strain analysis could discriminate between patients who later developed severe vs. moderate disease while still in the ED (area under the curve: 0.9167 (95% CI: 0.7481 to 1.0), p=0.0225).

Furthermore, left ventricular global longitudinal strain correlated strongly with whole blood TGF-β1 RNA levels in all COVID-19 patients (**Figure 2D**, Pearson-test), but particularly in patients who later developed severe disease (**Figure 2E**, Pearson-test). Of note, two of the patients with severe disease included in the blood and echocardiography analysis were later autopsied after death because of COVID-19 and were found to exhibit increased left ventricular fibrosis.

### Cardiac NRP-1 Expression Is Increased in Patients With Left Ventricular Fibrosis

Soluble IL1-RL1 has been shown to increase expression of NRP-1. Intriguingly, NRP-1 has been shown to facilitate the entry of SARS-CoV-2 into cells (18). We therefore hypothesized that increased cardiac fibrosis may predispose to severe progression of COVID-19 owing to increased NRP-1 expression in the heart enabling viral cell entry.

**Figure 3:**
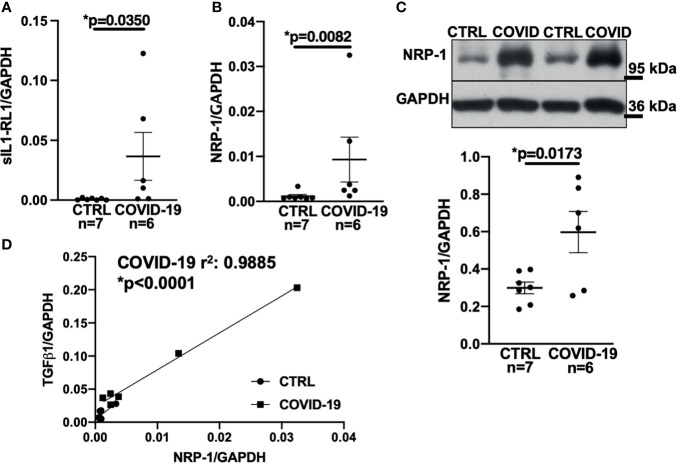
Expression of NRP-1 was significantly increased in the left ventricular myocardium of deceased COVID-19 patients and correlates with TGF-β1 mRNA levels. **(A)** Mean ± SEM mRNA levels (qPCR) of soluble interleukin-1 receptor-like 1 (sIL1-RL1) shows increased expression in left ventricular myocardium of deceased COVID-19 patients (Mann-Whitney test). **(B, C)** Mean ± SEM NRP-1 mRNA **(B)** and protein expression (**C,** original immunoblot in upper panel). Compared to CTRL, NRP-1 expression was significantly increased in the left ventricular myocardium of deceased COVID-19 patients both at mRNA (Mann-Whitney) and protein level (Student’s t-test). **(D)** Correlation analysis of left ventricular mRNA expression of TGF-β1 and NRP-1 (line indicates linear regression). There was a strong and significant correlation between NRP-1 and TGF-β1 mRNA.

Accordingly, sIL1-RL1 and NRP-1 RNA levels were significantly increased in the left ventricular tissue of patients who had died from COVID-19 compared to controls (**Figures 3A, B** respectively). We also confirmed this increased NRP-1 expression in the left ventricular tissue of COVID-19 patients by Western blotting (**Figure 3C**).

Consistent with these findings, NRP-1 RNA levels in the left ventricle correlated strongly with TGF-β1 levels (**Figure 3D**).

### Pulmonary NRP-1 Levels Correlate With SARS-CoV-2 Infection of the Lung

COVID-19 is primarily considered to be a pulmonary disease. Interestingly, TGF-β1 mRNA levels in the blood and left ventricular global longitudinal strain accurately predicted disease severity in our COVID-19 patients. We therefore hypothesized that increased systemic TGF-β1 expression, as seen in the blood samples taken from patients with severe cardiac fibrosis, might also predispose to viral infection of the lung due to increased pulmonary expression of NRP-1, which facilitates SARS-CoV-2 cell entry.

**Figure 4:**
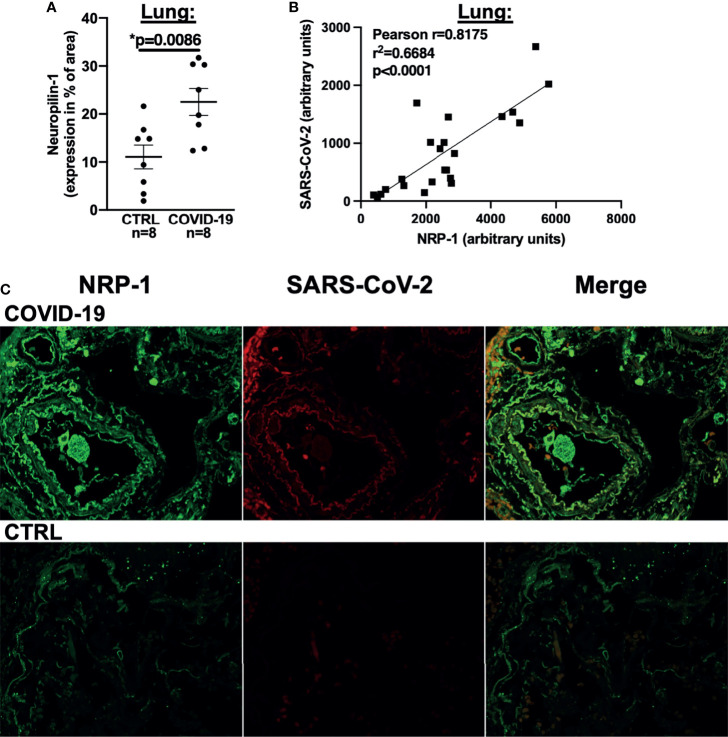
NRP-1 expression is increased in the lungs of deceased COVID-19 patients and correlates with the amount of SARS-CoV-2 nucleocapsid. **(A)** Mean ± SEM NRP-1 expression (as % of area) measured by immunofluorescence in lung sections. Compared to control, there was a significant increase in NRP-1 immunofluorescence in the lungs of deceased COVID-19 patients (Student’s t-test). **(B)** Correlation analysis with linear regression of NRP-1 and SARS-CoV-2 nucleocapsid immunofluorescence in lung sections. There was a strong and significant correlation between the amounts of NRP-1 and SARS-CoV-2 nucleocapsid detected in the lungs of the deceased COVID-19 patients. **(C)** Original immunofluorescence stainings of NRP-1 and SARS-CoV-2 (and merged immunofluorescence channels) in lung sections from a patient with severe COVID-19 and CTRL (representing the full region of interest used for analysis).

We thus performed NRP-1 immunohistochemical staining of lung tissue from our autopsied cohort with an antibody directed against NRP-1 and demonstrated that NRP-1 expression (positive staining as % of area) was drastically increased in patients who died of COVID-19 (**Figure 4A**). We then performed immunofluorescence staining of lung tissue with antibodies directed against NRP-1 and SARS-CoV-2 nucleocapsid. In COVID-19 patients, our analysis showed a clear correlation between pulmonary SARS-CoV-2 nucleocapsid fluorescence as a marker of SARS-CoV-2 viral infection and NRP-1 fluorescence (correlation in **Figure 4B**, original immunofluorescence stainings in **Figure 4C**). As expected, no relevant SARS-CoV-2 nucleocapsid signal was detected in the control patients.

## Discussion

This study demonstrates that patients who have died from COVID-19 have increased myocardial fibrosis. We postulate that this myocardial fibrosis is likely to be a pre-existing risk factor for severe disease courses of COVID-19, whereby increased expression of sIL1-RL1 induces NRP-1 expression that facilitates ACE2-mediated SARS-CoV-2 entry into the lung.

Direct cardiac effects of COVID-19 have been repeatedly demonstrated (1–3), including *de novo* heart failure and arrhythmias (37). Elevated cardiac troponin has been show to predict disease progression (38), yet the exact cause of myocardial injury in COVID-19 remains elusive.

Myocardial fibrosis has been detected by MRI in numerous patients with active SARS-CoV-2 infection and also in survivors of COVID-19 (6, 7). However, it is unknown whether cardiac fibrosis is caused by SARS-CoV-2 infection or merely a risk factor for severe disease courses that require patient hospitalization. In our study, SARS-CoV-2 virus could not be detected in myocardium taken from deceased COVID-19 patients. Other autopsy studies have found SARS-CoV-2 infection in cardiomyocytes and, in some cases, active myocarditis, but this has been shown to be a rare event (22, 23). We observed a significant correlation between the relative size of the fibrotic area and the number of CD3-positive cells if all control and COVID-19 sections were analyzed together (**Supplementary Figure 2A**). This is consistent with the fact that fibrosis is associated with some degree of inflammation. However, even in the severely diseased COVID-19 patients, the extent of inflammation was so low that it would not have been detectable macroscopically or by MRI. In any case, this minimal increase in CD3 staining does not warrant a diagnosis of myocarditis. Massive myocyte death could lead to myocardial infiltration of immune cells. While troponin values were increased in COVID-19, troponin did not correlate with either fibrosis (**Supplementary Figure 2B**) or CD3-positive cells (**Supplementary Figure 2C**). There are several explanations for this discrepancy. Firstly, the time points of blood collection and histological analysis were not synchronized. The former was performed upon hospital admission (early time point), whereas the latter occurred after patient death, usually several weeks later. Therefore, any immune cell infiltration in response to acute myocyte death may have already subsided. Secondly, the level of troponin was quite moderate and much lower than levels associated with, for example, myocardial infarction. A similar moderate increase in cardiac troponin is often observed in tachycardia due to atrial fibrillation or in increased afterload due to pulmonary embolism; these conditions are generally not associated with massive myocardial inflammation. Thirdly, patients were treated with corticosteroids for several weeks, which may have prevented immune cell infiltration and possibly viral entry. This could also be in line with previous studies, where inflammation warranting the diagnosis of myocarditis was limited to patients receiving non-steroidal immunosuppression (23), while patients on steroids, similar to those our study, showed no cases of myocarditis (23). It is also worth noting that current NIH guidelines recommend steroid treatment for all cases of severe COVID-19 (39). This means that the sample of patients in our study is highly representative of clinical reality, adhering to current therapy guidelines. Our dataset is therefore highly relevant and our findings can be extrapolated to COVID-19 patients seen in clinical settings.

In this publication, we report a high incidence of myocardial fibrosis in deceased COVID-19 patients. We also studied patients who were newly admitted to the ED because of COVID-19. At this early stage of disease, left ventricular function and clinical parameters did not identify patients who would later develop severe COVID-19. However, increased left ventricular strain as a marker of fibrosis accurately predicted moderate and severe disease courses. This may indicate that significant pre-existing cardiac fibrosis was already present at initial presentation to the ED and thus at an early stage of disease when SARS-CoV-2-induced fibrosis is at least highly unlikely. Differences in ejection fraction between moderate and severe cases of COVID-19 and control patients were small, despite the fact that severe COVID-19 patients had marked left ventricular fibrosis. Left ventricular fibrosis is also a hallmark of heart failure with preserved ejection fraction (HFpEF) (40–42) and the latter may have remained undetected in our patient population. The decreased global longitudinal strain in our study might be indicative of cardiac fibrosis rather than systolic contractile dysfunction.

TGF-β1 and reactive oxygen species generated *via* NOX2 and NOX4 are significant inducers of cardiac fibrosis (13, 15, 24), but also regulate inflammation (43). Furthermore, sIL1-RL1 can induce TGF-β1 expression and cardiac fibroblast activation (12). Interestingly, TGF-β1 mRNA levels were significantly increased in the blood of patients who presented to the ED and later developed severe disease. Furthermore, these levels correlated strongly with left ventricular myocardial strain.

Profibrotic induction of SMAD3, NOX2 and NOX4 was also present at this early stage in patients who would later develop severe disease.

The cellular sources of TGF-β1 are undoubtedly abundant. Besides TGF-β1 produced by tissue-derived fibrocytes [e.g (15, 25, 28, 29)], cardiac and renal fibrosis may also be regulated by platelet-derived TGF-β1 RNA (44). This non-cardiac source has been shown to contribute to cardiac dysfunction (45). However, the source of TGF-β1 in fibrotic organs remains poorly defined and may also depend on other cell types such as macrophages and T-cells, as well as the extracellular matrix (46). Regardless of the source, the marked differences between the severe COVID-19 patients and control subjects in terms of blood and cardiac TGF-β1 expression and the strong correlation of blood TGF-β1 RNA with cardiac strain suggest an important role for TGF-1 in the development of severe COVID-19.

sIL1-RL1 has been shown to increase NRP-1 expression in adult human cardiac fibroblasts and both receptors are increased in murine cardiomyocytes from a model of pressure overload (12). Moreover, NRP-1 facilitates the entry of SARS-CoV-2 into human host cells (18). In myocardium of deceased COVID-19 patients, we detected an elevated level of NRP-1 mRNA and protein expression that correlated highly with TGF-β1 expression. Considering the absence of heart failure prior to death in the autopsied population and our observation that fibrosis and increased TGF-β1 RNA levels accurately predicted disease severity in COVID-19 patients presenting to the ED, we hypothesize that increased systemic TGF-β1 expression might predispose patients to viral infection of the lung due to increased pulmonary expression of NRP-1 as a SARS-CoV-2 entry receptor. Consistent with this, we demonstrated significant upregulation of pulmonary NRP-1 expression in our autopsied group, which directly correlated with the levels of SARS-CoV-2 nucleocapsid detection. Our data may also have implications for other organ systems, such as the kidneys or the liver, for which NRP-1 and TGF-β1 expression has either been directly implicated in SARS-CoV-2 infection (47) or is involved in organ fibrosis (13).

A limitation of this study is that myocardial biopsies from patients with mild to moderate disease could not be included for ethical reasons. In addition, increased myocardial strain - although a strong indicator of myocardial fibrosis when left ventricular ejection fraction is normal - is of course only an indirect measure of cardiac fibrosis. The financial expense of detecting fibrosis *via* cardiac MRI in patients with severe disease (i.e. intubated and on vasopressor support) was a limiting factor. For the patients with histological data, no macroscopic or histological evidence of myocarditis was found in any of the heart specimens, however, it is not possible to investigate the entire heart histologically. Therefore, we cannot completely exclude the presence of macroscopically invisible focal myocarditis, although we acknowledge that this is very unlikely. The discrepancy between the clear correlation of NRP-1 expression and pulmonary SARS-CoV-2 infection on the one hand and the absence of SARS-CoV-2 virus in our cardiac samples on the other hand, may point towards technical limitations of cardiac SARS-CoV-2 detection but may also be a result of the aforementioned corticosteroid treatment. Furthermore, the abundant expression of NRP-1 on the large surface area of the lungs (18) may also enable prolonged SARS-CoV-2 detection.

**Figure 5:**
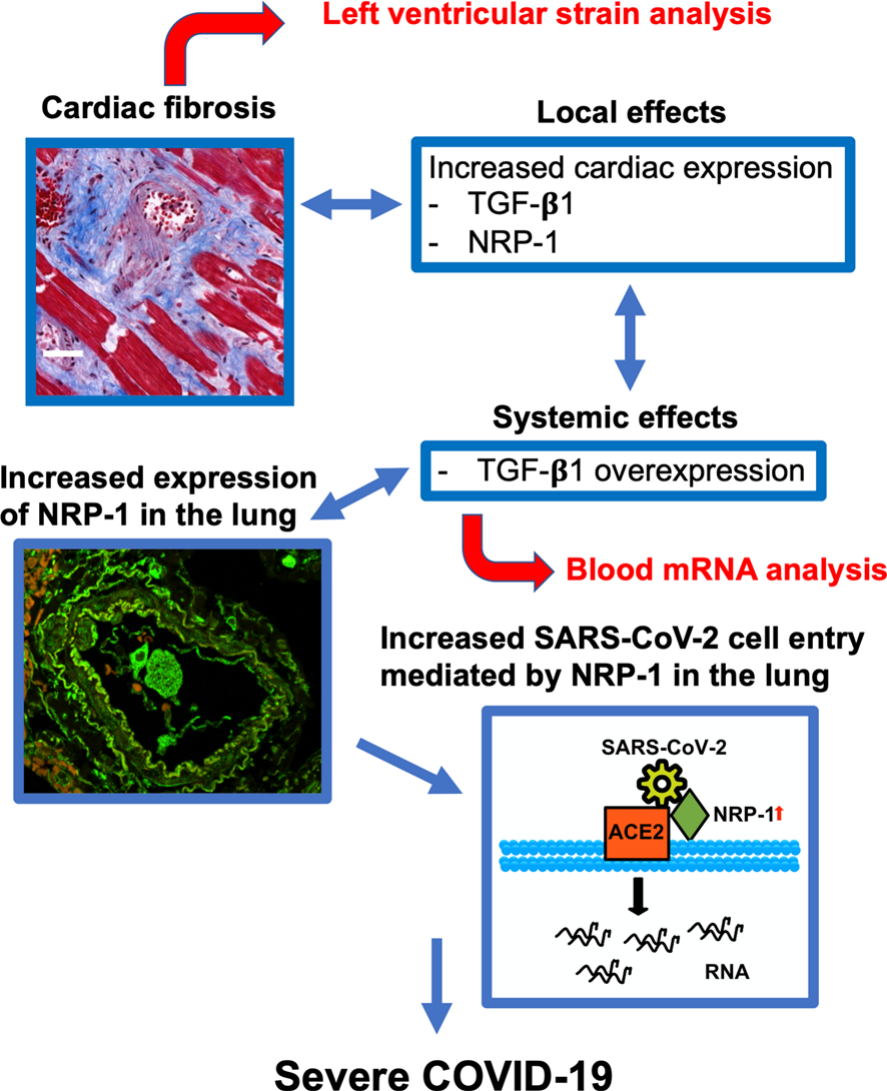
Predictive model for severe COVID-19. Cardiac fibrosis may predispose COVID-19 patients to severe disease courses due to increased cardiac expression and systemic overexpression of TGF-β1. Elevated expression of NRP-1 in fibrotic tissue can facilitate cell entry of SARS-CoV-2 *via* ACE2 and thereby promote viral infectivity, leading to severe disease courses of COVID-19. Risk stratification can be performed in newly hospitalized patients using echocardiographic strain imaging and qPCR analysis of whole blood TGF-β1 mRNA.

In summary, our data suggest that cardiac fibrosis is a risk factor for severe disease progression (**Figure 5**). Increased systemic expression of TGF-β1 (and sIL1-RL1) may promote pulmonary entry of SARS-CoV-2 owing to increased expression of NRP-1, which facilitates SARS-CoV-2 cell entry. Cardiac strain and whole blood TGF-β1 mRNA analysis can detect fibrosis in patients presenting to the ED and may potentially allow early and efficient risk stratification in this clinical context.

## Data Availability Statement

Original data can be made available in a blinded manner upon reasonable request.

## Ethics Statement

The studies involving human participants were reviewed and approved by University Medical Center Regensburg. The patients/participants provided their written informed consent to participate in this study.

## Author Contributions

JM and SW designed the study, gathered and interpreted data, wrote the manuscript and are responsible for the integrity of the article. JH, MB, KB, CB, CJ, CM, and BS gathered and interpreted data and provided intellectual review of the article. ME and LM provided critical intellectual content of the manuscript and revised the final paper. All authors contributed to the article and approved the submitted version.

## Funding

JM is funded by the German Cardiac Society Clinician Scientist program and by the Deutsche Forschungsgemeinschaft (DFG, German Research Foundation) grant MU 4555/2-1 (project number 455425596). LM is funded by DFG grant MA1982/5‐1. SW and LM are funded by SFB 1350 TPA6 and the University of Regensburg ReForM C programme. SW is funded by DFG grants WA 2539/4‐1, 5‐1, 7‐1, and 8‐1. MB is supported by the German Heart Foundation/German Foundation of Heart Research – F/50/20. SW is funded by DFG grant WA 2539/8-1. SW and LM are also supported by the DFG SFB 1350 grant (Project Number 387509280, TPA6).

## Conflict of Interest

The authors declare that the research was conducted in the absence of any commercial or financial relationships that could be construed as a potential conflict of interest.

## Publisher’s Note

All claims expressed in this article are solely those of the authors and do not necessarily represent those of their affiliated organizations, or those of the publisher, the editors and the reviewers. Any product that may be evaluated in this article, or claim that may be made by its manufacturer, is not guaranteed or endorsed by the publisher.

## Supplementary Material

Supplementary Figure 1 Left ventricular fibrosis is also increased in the subgroup of deceased patients with echocardiographic recordings. **(A)** Left ventricular ejection fraction does not differ significantly between COVID-19 patients who later died due to the disease and controls (mean ± SEM, Student’s t-test). **(B)** Histochemical analysis of left ventricular fibrotic tissue (trichrome) indicates an increased area of fibrosis in the subgroup of deceased patients with echocardiographic data (mean ± SEM, Student’s t-test). **(C)** Global longitudinal strain is - by trend - also increased (i.e. less negative) in the limited number of deceased COVID-19 patients with echocardiographic data available.

Supplementary Figure 2 No significant association of left ventricular fibrosis or immune cell infiltration with the extent of myocardial injury in COVID-19 and control patients **(A)** The extent of fibrosis (trichrome stainings) and CD3+ immunofluorescence were correlated in left ventricular tissue sections taken from COVID-19 and control patients. The relative size of the fibrotic area and percentage of CD3-positive cells correlated significantly, but only if all sections (controls and COVID-19) were analyzed together. **(B, C)** Massive myocyte death may lead to myocardial infiltration of immune cells. However, the extent of myocardial injury (blood troponin levels) does not significantly correlate with either the extent of ventricular fibrosis **(B)** or the percentage of CD3-positive cells **(C)** (lines show linear regression).
