# Supplementary figures and images for: Cardiac Fibrosis Is a Risk Factor for Severe COVID-19
Source: Front Immunol. 2021 Oct 22;12:740260. doi: 10.3389/fimmu.2021.740260 (PMC8569622; doi:10.3389/fimmu.2021.740260)

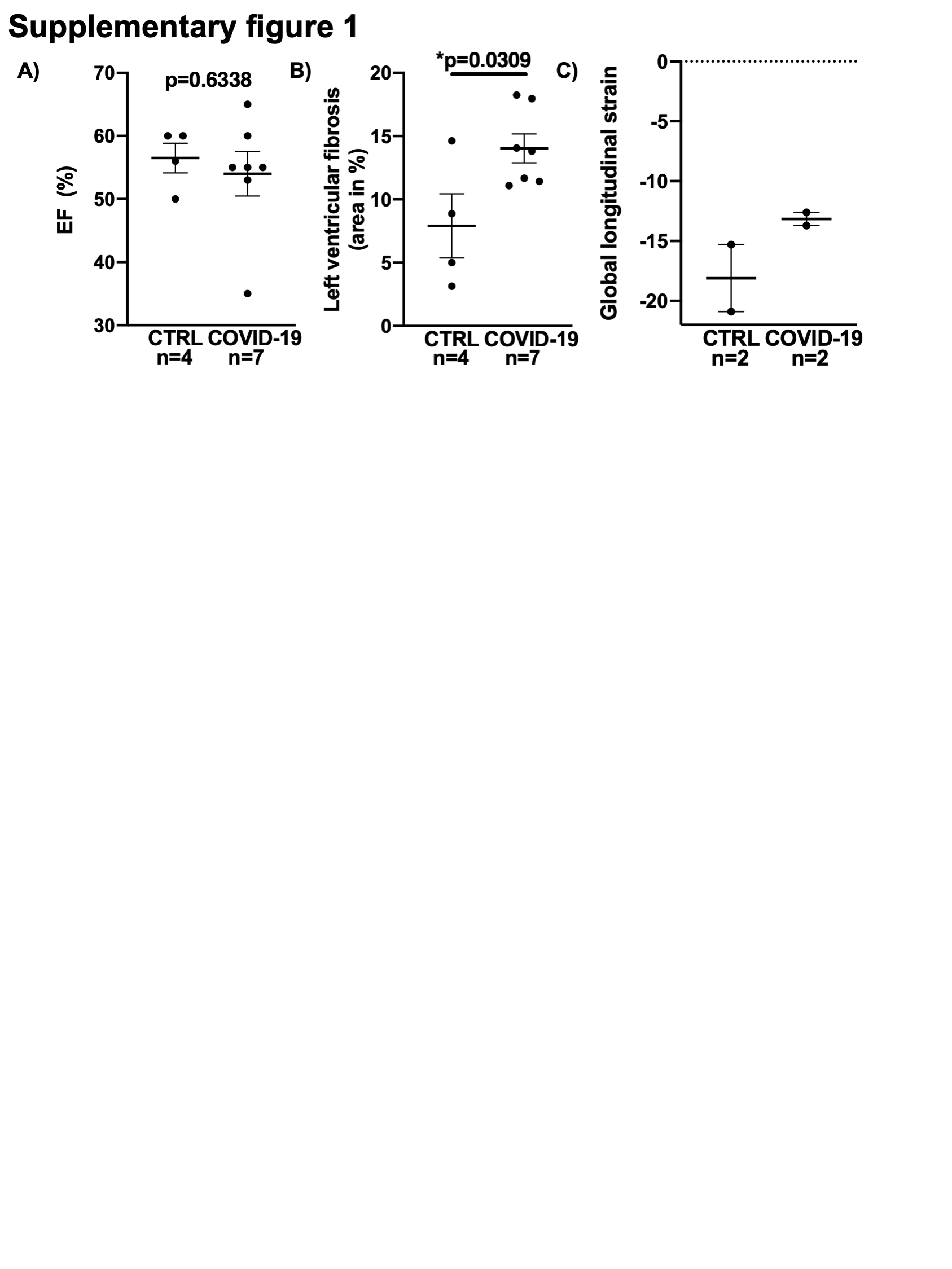

Supplement: Supplementary Figure 1 — Left ventricular fibrosis is also increased in the subgroup of deceased patients with echocardiographic recordings. (A) Left ventricular ejection fraction does not differ significantly between COVID-19 patients who later died due to the disease and controls (mean ± SEM, Student’s t-test). (B) Histochemical analysis of left ventricular fibrotic tissue (trichrome) indicates an increased area of fibrosis in the subgroup of deceased patients with echocardiographic data (mean ± SEM, Student’s t-test). (C) Global longitudinal strain is - by trend - also increased (i.e. less negative) in the limited number of deceased COVID-19 patients with echocardiographic data available. [file Image_1.tiff]

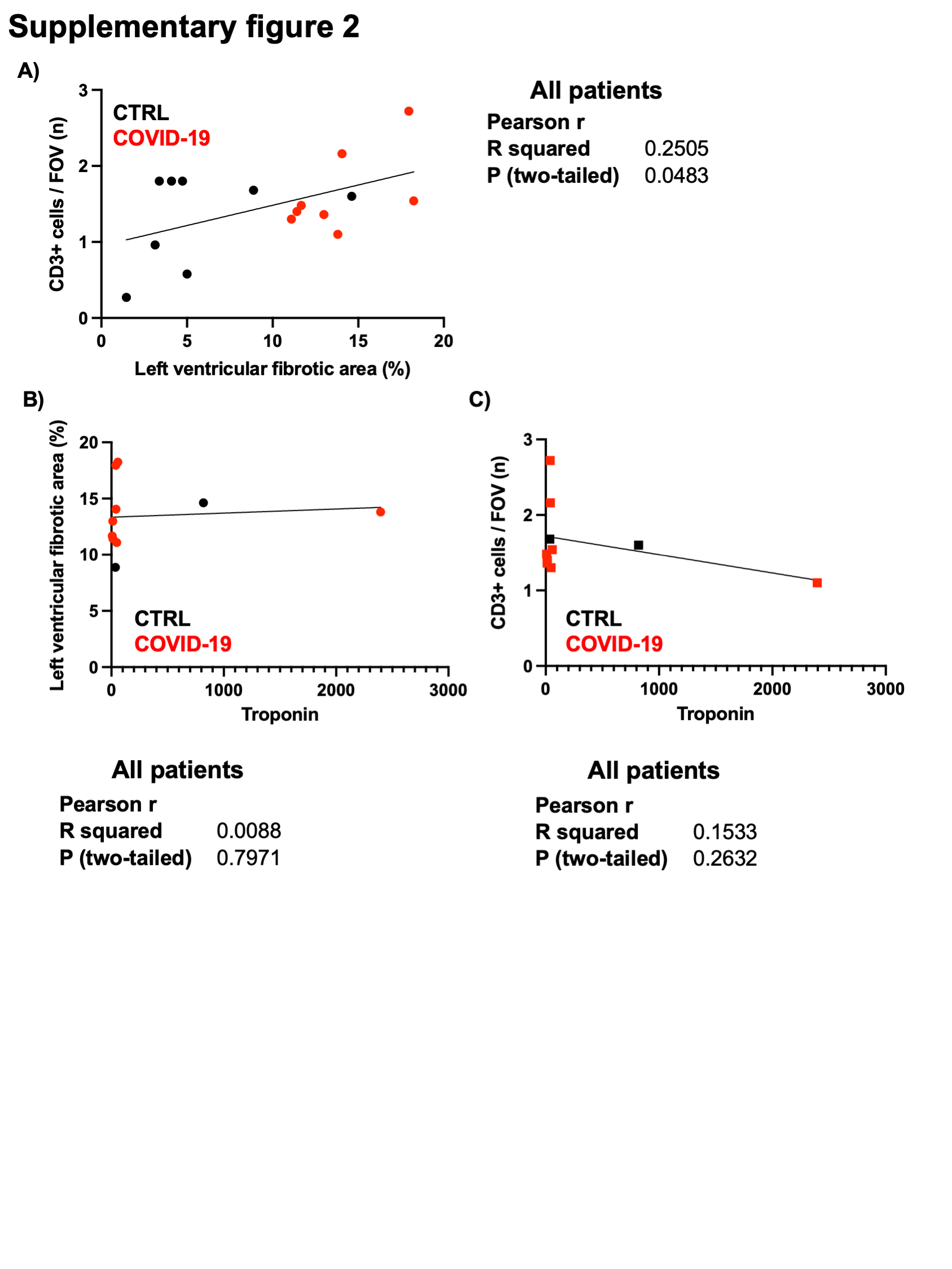

Supplement: Supplementary Figure 2 — No significant association of left ventricular fibrosis or immune cell infiltration with the extent of myocardial injury in COVID-19 and control patients (A) The extent of fibrosis (trichrome stainings) and CD3+ immunofluorescence were correlated in left ventricular tissue sections taken from COVID-19 and control patients. The relative size of the fibrotic area and percentage of CD3-positive cells correlated significantly, but only if all sections (controls and COVID-19) were analyzed together. (B, C) Massive myocyte death may lead to myocardial infiltration of immune cells. However, the extent of myocardial injury (blood troponin levels) does not significantly correlate with either the extent of ventricular fibrosis (B) or the percentage of CD3-positive cells (C) (lines show linear regression). [file Image_2.tiff]
